# Low Generation *Culex quinquefasciatus* from Texas and *Culex pipiens* from New Jersey Are Incompetent Vectors for Oropouche Virus

**DOI:** 10.4269/ajtmh.26-0251

**Published:** 2026-09-10

**Authors:** Erik Turner, Nicole Foley, Casey Parker-Crockett, Linda Kothera, Emily Davis, Matylda Lally, Cassandra Durden, Joan L. Kenney

**Affiliations:** ^1^ Division of Vector Borne Disease, Centers for Disease Control and Prevention, Fort Collins, Colorado, USA;; ^2^ Oak Ridge Institute for Science and Education, Oak Ridge, Tennessee, USA

## Abstract

During the 2024 Oropouche virus (OROV) outbreak in South America, mosquito surveillance led to the identification of pools of *Culex quinquefasciatus* infected with the virus. Because of the outbreak strain’s status as a novel reassortment and the novel sequela discovered during this outbreak, concerns arose about *Culex* species as potential vectors in the continental United States. To assess the risk posed by *Cx. quinquefasciatus* and the closely related species *Culex pipiens*, the vector competence of mosquitoes reared from eggs collected in Dallas, TX, and New Jersey was examined. Three replicates of an infectious bloodmeal were offered to *Cx. quinquefasciatus* at generations F2, F4, and F9, resulting in 0/305 individuals with midgut infections. One infectious bloodmeal was offered to *Cx. pipiens* at generation F4, with 0/120 resulting midgut infections. These data, which align with other recent publications, indicate that United States populations of *Cx. quinquefasciatus* and *Cx. pipiens* are not competent vectors of the outbreak strain of OROV.

## INTRODUCTION

Oropouche virus (OROV), first isolated from a human in Trinidad and Tobago in 1955,^1^ is endemic to South America in the Amazonian region and causes focal sporadic outbreaks, primarily in Brazil.^2^ However, it is generally considered an understudied arbovirus with nonspecific symptoms that mirror other circulating diseases in humans.^3^ In late 2023, the number of human OROV cases in Brazil spiked and was associated with a newly recognized reassortant OROV strain. Over the next year, 16,239 confirmed human cases were observed in Brazil, Bolivia, Colombia, Ecuador, Peru, Cuba, Guyana, Barbados, and Panama.^4^^–^^6^ In 2025, an additional 13,094 confirmed cases were reported, with cases also being identified in Venezuela and Uruguay.^7^ During 2024, cases associated with travel to the affected areas were identified in seven states in the United States, with the majority (>95%) of infected individuals living in Florida.^8^ Given the intensity, rapid geographic expansion of the outbreak, and documented neurological symptoms and vertical transmission associated with microcephaly and fetal abortions, understanding the transmission risk was considered an urgent concern.^9^^,^^10^

The primary vector of OROV is reported to be *Culicoides paraensis*,^11^ a biting midge with a widespread distribution in the Americas. However, at the beginning of the 2024 outbreak, mosquitoes, particularly *Culex quinquefasciatus*, were considered potential secondary vectors in Brazil because of the abundance of infected pools identified.^6^^,^^12^ Similarly, surveillance studies in epidemic areas of Brazil revealed relatively high numbers of OROV-positive *Cx. quinquefasciatus* pools, which has contributed to the postulation that *Cx. quinquefasciatus* may serve as secondary urban vectors.^12^^,^^13^ Furthermore, the active OROV transmission in Cuba, where *Cu. paraensis* had yet to be recorded, supported this idea.^14^^,^^15^ To date, this theory is not supported by existing experimental data on vector competence in *Culex* species. Experiments involving oral exposure of OROV virus to *Culex* species have revealed a very low transmission potential, with multiple publications reporting no detected transmission capability.^16^^–^^23^

Many vector competence studies involve the use of well-established mosquito colonies because they are easy to work with, readily available, and have historical data on vector competence for other viruses. However, using these colony mosquitoes for vector competence studies presents challenges. For instance, many colonies were established years ago and are inbred, which does not reflect the genetics of wild populations.^24^ Additionally, the microbiome of these colony mosquitoes does not represent field conditions.^25^ Therefore, although colony mosquitoes are an important tool for establishing vector competence potential, the results from these studies might not represent the competence status of field populations of mosquitoes. Furthermore, geographically distinct field populations frequently exhibit significant variation in vector competence.^26^^,^^27^

Unlike West Nile virus and Saint Louis encephalitis virus, which are transmitted by *Culex pipiens* and *Cx. quinquefasciatus*, OROV generates sufficient viremia in human blood and can be acquired by mosquitoes for subsequent transmission to naïve hosts. This increases the risk of an autochthonous urban human/mosquito transmission cycle. Additionally, because of the continued proliferation of literature identifying *Cx. quinquefasciatus* as a secondary vector solely on the basis of identification of infected pools in the field,^12^^,^^13^ it is important to assess its potential for transmission competence. The vector competence of U.S. field-derived low-generation *Cx. quinquefasciatus* and *Cx. pipiens* for the circulating strain of OROV was examined in the present study to help establish the risk of local OROV transmission in the continental United States.

## MATERIALS AND METHODS

### Egg raft sources.

*Culex quinquefasciatus* egg rafts were collected from bins of water by collaborators (Dallas County Health and Human Services) in the Dallas/Fort Worth area, TX, United States, in October 2024. These were shipped overnight to the CDC in Fort Collins, Colorado, with each raft individually packaged on moistened filter paper in a microcentrifuge tube and kept on ice. Egg rafts were reared individually in 100 × 25 mm plastic Petri dishes, with 65 mg of liver powder and 1 mg of ground fish food, and dishes were filled with 60 mL of deionized water. Hatched larvae were monitored and given liver powder as needed. *Culex pipiens* were also collected by collaborators in Vector Control districts in New Jersey, United States (Mercer County Mosquito Control), from two locations. These were reared as described above.

### Mosquito identification and rearing.

Larvae identification was performed under a dissection microscope on fourth-instar larvae using a taxonomic key.^28^ Larvae were reared to adults on a diet of liver powder and fish food at 28°C in environmental chambers with ∼75% humidity. Pupae were removed and placed in containers within mosquito cages and offered 5% sucrose solution ad libitum via gauze-covered small glass jars. Meals of uninfected defibrinated chicken blood were offered to adult mosquitoes at 7 to 10 days post-emergence. Mosquitoes were sugar-starved for 12 hours with deionized water available. The bloodmeal was offered overnight via a Hemotek (Hemotek Ltd., Blackburn, United Kingdom) reservoir covered with Parafilm^®^ (Amcor, Zurich, Switzerland) and kept at 37°C. Four days post-blood meal, water in a Petri dish was introduced into the cage. Two types of egg-laying water were used in the present study: distilled water and a hay water solution produced by boiling feed-grade hay as described by Bhujel and Saha.^29^ Pools of F1 adults were subjected to de novo next-generation sequencing to rule out the presence of pathogenic viruses.

### Virus source.

The OROV strain 240023 (GenBank # PQ41798; PQ417949; PQ417950) used in these experiments was isolated from a human travel case identified in Florida. The isolate was obtained from the Arbovirus Reference Collection, and it had a history of two passages in Vero cells. The virus was passaged once more on Vero cells to generate a working viral stock of 6.8 log_10_ (PFU/mL), which was used as the starting material for both frozen stock feeds and cell inoculation for active virus challenges. Vero cell-based plaque assays were used to titrate stock strains and experimental bloodmeals, as previously described.^30^

### Artificial bloodmeal exposure.

Three different bloodmeal replicates were used in the present study, each offered via Hemotek blood feeders at 37°C. The first composition (BM1), which was offered to F2 and F4 *Cx. quinquefasciatus* and F4 *Cx. pipiens*, consisted of 2 mL of chicken blood (Rockland Immunochemicals, Limerick, PA, USA), 0.5 mL of thawed virus stock, and 0.5 mL of a 10% sucrose–fetal bovine solution per Hemotek disk. The second bloodmeal composition (BM2) was the same, apart from the thawed viral stock, which had a titer of 7.3 log_10_ (PFU/mL). The third bloodmeal (BM3) contained 2.5 mL of chicken blood and 0.5 mL of fresh viral supernatant (OROV 230024 v4), collected 60 hours post-inoculation (MOI 0.1), per Hemotek feeder. For each bloodmeal, 1 mL of the bloodmeal composite was collected to start titer quantification.

Before each bloodmeal, mosquitoes were transferred to six cartons and sugar-starved for 12 hours (deionized water replaced sugar water to help prevent desiccation). BM1 was offered for 24 hours (9:00–9:00), BM2 was offered for 12 hours (20:00–8:00), and BM3 was offered for 10 hours (21:00–7:00). The duration of blood meals was dependent on observed feed rates. Upon completion of each bloodmeal, 1 mL of the remaining composite in each Hemotek disk was collected for ending titer quantification. Mosquitoes were then cold anesthetized at −20°C, sorted by fed status, and maintained at 28°C with 5% sucrose ad libitum via cotton balls.

### Vector competence analysis.

#### Mosquito dissection.

At 7, 14, and 21 days post-exposure (dpe), mosquitoes were cold anesthetized, and then their legs and wings were dissected and collected into microcentrifuge tubes with complete Dulbecco's Modified Eagle Medium (DMEM) and a copper-coated BB. Saliva was then collected by inserting proboscises into micropipette tips filled with a 10% sucrose in fetal bovine serum (FBS) solution for 30 minutes. The solution was then added to complete DMEM, and the bodies were collected as described for the legs and wings. The legs, wings, and bodies were then ground using a Thermo Fisher Tissuelyser (Thermo Fisher Scientific, Waltham, MA) for 3 minutes at 25 Hz and then centrifuged at 10,000 rpm at 8°C for 5 minutes before storage at −80°C.

#### RNA extraction and quantitative reverse transcription polymerase chain reaction.

MagMAX Viral/Pathogen 96 extraction kits (Thermo Fisher Scientific) were used to extract RNA from the samples, utilizing the Kingfisher extraction platform per manufacturer instructions. Two hundred *µ*L of each sample was used, and RNA was eluted into 50 *µ*L. A standard curve of OROV 230024 was extracted along with the samples and negative extraction controls. The QuantiTect Virus Quantitative Reverse Transcription Polymerase Chain Reaction (qRT-PCR) kit (QIAGEN, Hilden, Germany) was used for qRT-PCR in accordance with the manufacturer’s instructions. Specifically, 2 *µ*L of sample RNA was used, with a 0.2 *µ*M concentration of primers and probes, as described by Naveca et al.^31^ (Table 1). The reverse transcription step was performed for 20 minutes at 50°C, halted at 95°C for 5 minutes, and then cycled between 95°C for 15 seconds and 60°C for 45 seconds for 45 cycles.

**Table 1:** Oropouche virus primers developed by Naveca et al., used for quantitative reverse transcription polymerase chain reaction testing

Primer	Sequence
Forward primer	5′-TCCGGAGGCAGCATATGTG-3′
Reverse primer	5′-ACAACACCAGCATTGAGCACTT-3′
Probe	5′(FAM) CATTTGAAGCTAGATACGG-3′

## RESULTS

### Colony development.

Colony development of *Culex* mosquitoes required multiple attempts. In the second attempt, *Cx. quinquefasciatus* from 23 field-collected egg rafts and *Cx. pipiens* from 33 field-collected egg rafts fed on chicken blood and laid 101 and 38 egg rafts, respectively. Boiled hay water and overnight feeding were required for successful propagation. To reach the numbers needed for vector competence, multiple generations were allowed to lay as many eggs as possible (two generations for *Cx. quinquefasciatus* and four generations for *Cx. pipiens*), up to a maximum of 240 rafts. Next-generation sequencing of F1 pools from each colony was used to rule out the presence of any pathogenic viruses, although putative insect-specific viruses were identified (data not shown).

### Vector competence.

Three different bloodmeal compositions were offered to F2, F4, and F9 *Cx. quinquefasciatus*, and each was tested via qRT-PCR for midgut infection (Table 2). For BM1, 30 mosquitoes survived for analysis at both 7 and 14 dpe, and 35 mosquitoes survived and were tested at 21 dpe. After BM1, no mosquito bodies were positive for OROV RNA (Table 2). Because all test results for the initial infection were negative, testing was not conducted for dissemination or transmission. BM2 was created from a viral stock with a higher titer and yielded 35 mosquitoes at each testing time point; however, BM2 also resulted in no infected bodies (Table 2). For BM3, 50 individual mosquitoes survived and were tested at each time point: 7, 14, and 21 dpe. BM3 contained freshly sourced virus because evidence indicates that fresh virus can alter infectiousness in mosquitoes for some viruses^32^; however, no infected individuals were identified after this challenge (Table 2). Because BM2 and BM3 similarly did not yield any detected body infection, testing downstream dissemination and transmission was not continued for these replicates, and no virus titrations were attempted. In total, 305 *Cx. quinquefasciatus* were exposed and tested, and zero demonstrated oral infection. Additionally, BM1 was offered to F4 *Cx. pipiens*, and 40 individuals survived and were tested at 7, 14, and 21 dpe (120 total). No midgut infections were detected at any time point. Bloodmeals were not offered to subsequent generations of *Cx. pipiens*.

**Table 2:** Positive and negative quantitative reverse transcription polymerase chain reaction results from vector competence experiments at each time point

Bloodmeal	Mosquito	Generation	Virus Source	Feed Duration (hours)	Starting Bloodmeal Titer log_10_ (PFU/mL)	Ending Bloodmeal Titer Range log_10_ (PFU/mL)	Midgut/7 dpe	Midgut/14 dpe	Midgut/21 dpe
1	*Cx. quinquefasciatus*	F2	Frozen stock	24	5.4	4.8–4.4	0/30	0/30	0/35
1	*Cx. pipiens*	F4	Frozen stock	24	5.7	4.9–4.4	0/40	0/40	0/40
2	*Cx. quinquefasciatus*	F4	Frozen stock	12	5.9	5.4–4.0	0/35	0/35	0/35
3	*Cx. quinquefasciatus*	F9	Fresh virus	10	6.0	5.0–4.9	0/50	0/50	0/50

*Cx. pipiens* = *Culex pipiens*; *Cx. quinquefasciatus* = *Culex quinquefasciatus*; dpe = days post-exposure; PFU = plaque forming units.

## DISCUSSION

The widespread outbreak of OROV throughout the Americas in 2024 resulted in the identification of multiple travel cases in the United States. Previous implications of *Cx. quinquefasciatus* as a potential secondary vector and the ability of OROV to cause human viremia raised concerns about the establishment of autochthonous transmission. To address these concerns, low-passage colonies of *Cx. quinquefasciatus* from Dallas, TX, and *Cx. pipiens* from New Jersey were established and orally challenged with an OROV strain isolated during the outbreak. These efforts indicate that the *Cx. quinquefasciatus* population from Dallas and the *Cx. pipiens* population from New Jersey are not competent vectors for OROV and thus are unlikely to contribute to virus establishment and autochthonous transmission.

Other examinations of *Culex* species vector competence with this strain of OROV have revealed similar results, with most colonies exhibiting the inability to transmit, although some have exhibited low proportions of midgut infection and disseminated infections.^19^^–^^21^^,^^23^ Only two studies have revealed transmission in *Culex* species (one *Cx. quinquefasciatus* individual from a 2014 Florida colony with unknown generation history and one F50 *Cx. pipiens* originating from New York in 2022), as indicated by the presence of OROV RNA in the saliva. In both examples, positivity was identified in a single specimen, with a 2% positivity rate for *Cx. pipens* and a 0.7% positivity rate for *Cx. quinquefasciatus*, and the presence of live virus was confirmed in only the *Cx. quinquefasciatus* specimen.^19^^,^^22^ Together with the low-generation *Culex* colonies, the current data highlight that U.S. *Cx. quinquefasciatus* and *Cx. pipiens* are poor vectors of OROV. Furthermore, because the study experiments resulted in no detected infection despite multiple attempts, these field populations may be refractory and thus less susceptible than some colonized *Cx. quinquefasciatus* and *Cx. pipiens,* highlighting the importance of nuanced investigations into vector competence.

During the peak of the 2024 OROV outbreak, health organizations cited *Cx. quinquefasciatus* as a potential secondary vector in disseminated information.^4^ This may have affected outbreak control and preparation in multiple ways, including vector control efforts being diverted from *Cu. paraensis.* Additionally, including *Cx. quinquefasciatus* ranges in predictive models to identify zones of potential transmission risk would likely result in inaccurate risk maps. Accurately informing the public of their risks and the causative vector of an outbreak is an important aspect of limiting transmission and preventing disease. However, because distinct mosquito populations have differing levels of competence for the same virus, it cannot be ruled out that other *Cx. quinquefasciatus* or *Cx. pipiens* populations could act as meaningful secondary OROV vectors.^26^^,^^27^ However, given the multiple instances of low to no competence in different established colonies, as well as a low-generation field-derived population, it seems highly unlikely that this species has or will contribute meaningfully to OROV transmission in the United States.

## CONCLUSION

Although laboratory vector competence studies provide valuable insight into the risk of autochthonous transmission, vectorial capacity is complex and not fully addressed in studies such as these. Mosquito density and biting rates may increase the risk even when very low vector competence may be of concern. However, North American *Cx. quinquefasciatus* and *Cx. pipiens* are primarily ornithophilic, which would diminish the risk of OROV transmission to humans.^33^ To bolster the data supporting the poor competence of *Culex* species for OROV and better understand the differences between populations, more vector competence studies with field-generated colonies from a wide geographic range are warranted. Additionally, using vectorial capacity models to assess the thresholds required for poor vectors to contribute to the establishment of autochthonous transmission may be of value in future studies.

## References

[1] Anderson CR Spence L Downs WG Aitken TH, 1961. Oropouche virus: A new human disease agent from Trinidad, West Indies. Am J Trop Med Hyg 10: 574–578. 10.4269/ajtmh.1961.10.574 13683183

[2] Sakkas H Bozidis P Franks A Papadopoulou C, 2018. Oropouche fever: A review. Viruses 10(4): 175. 10.3390/v10040175 PMC5923469 29617280

[3] Fonseca L Carvalho RH Bandeira AC Sardi SI Campos GS, 2020. Oropouche virus detection in febrile patients’ saliva and urine samples in Salvador, Bahia, Brazil. Jpn J Infect Dis 73(2): 164–165. 10.7883/yoken.JJID.2019.296 31787741

[4] Pan American Health Organization, 2025. *Epidemiological Update Oropouche in the Americas Region – 11 February 2025*. Available at: https://www.paho.org/en/documents/epidemiological-update-oropouche-americas-region-11-february-2025. Accessed August 21, 2026.

[5] Castro MC Lima Neto AS, 2024. Unprecedented spread and genetic evolution of the Oropouche virus. Nat Med 30(12): 3420–3421. 10.1038/s41591-024-03336-5 39468365

[6] Bai F Denyoh PMD Urquhart C Shrestha S Yee DA, 2025. A comprehensive review of the neglected and emerging Oropouche virus. Viruses 17(3): 439. 10.3390/v17030439 PMC11945866 40143366

[7] Pan American Health Organization, 2026. *ARBO Portal – Oropouche*. Available at: https://www.paho.org/es/arbo-portal/arbo-portal-oropouche. Accessed August 21, 2026.

[8] Centers for Disease Control and Prevention, 2025. *Oropouche in the United States*. Available at: https://www.cdc.gov/oropouche/data-maps/current-year-data.html. Accessed August 21, 2026.

[9] Schwartz DA Dashraath P Baud D, 2024. Oropouche virus (OROV) in pregnancy: An emerging cause of placental and fetal infection associated with stillbirth and microcephaly following vertical transmission. Viruses 16(9): 1435. 10.3390/v16091435 PMC11437435 39339911

[10] Guagliardo SAJ Connelly CR Lyons S Martin SW Sutter R Hughes HR Brault AC Lambert AJ Gould CV Staples JE, 2024. Reemergence of Oropouche virus in the Americas and risk for spread in the United States and its territories, 2024. Emerg Infect Dis 30(11): 2241–2249. 10.3201/eid3011.241220 PMC11521165 39353409

[11] Pinheiro FP Hoch AL Gomes ML Roberts DR, 1981. Oropouche virus. IV. Laboratory transmission by *Culicoides paraensis*. Am J Trop Med Hyg 30(1): 172–176. 7212164

[12] Feitoza LHM, , 2025. Integrated surveillance for Oropouche virus: Molecular evidence of potential urban vectors during an outbreak in the Brazilian Amazon. Acta Trop 261: 107487. 10.1016/j.actatropica.2024.107487 39622307

[13] Cardoso BF Serra OP Heinen LB Zuchi N Souza VC Naveca FG Santos MA Slhessarenko RD, 2015. Detection of Oropouche virus segment S in patients and in *Culex quinquefasciatus* in the state of Mato Grosso, Brazil. Mem Inst Oswaldo Cruz 110(6): 745–754. 10.1590/0074-02760150123 PMC4667577 26517653

[14] Dunford JC, , 2026. A review and update of the distribution, bionomics, and medical importance of *Culicoides (Haematomyidium) paraensis* (Diptera: Ceratopogonidae) in the United States in response to recent Oropouche virus expansion in the Americas. J Med Entomol 63(1): tjag019. 10.1093/jme/tjag019 41760083

[15] Pérez YM Ibáñez AC Díaz ZM Acosta EC González MS García NC Del Rosario Casanova Drake Q Gutierrez-Bugallo G, 2025. First report of *Culicoides paraensis* (Goeldi, 1905) (Diptera: Ceratoponidae) in Cuba: A new challenge for public health. Parasite Epidemiol Control 29: e00423. 10.1016/j.parepi.2025.e00423 PMC11995114 40230829

[16] Hoch AL Pinheiro FP Roberts DR Gomes ML, 1987. Laboratory transmission of Oropouche virus by *Culex quinquefasciatus* Say. Bull Pan Am Health Organ 21(1): 55–61. 3607353

[17] de Mendonça SF Rocha MN Ferreira FV Leite T Amadou SCG Sucupira PHF Marques JT Ferreira AGA Moreira LA, 2021. Evaluation of *Aedes aegypti, Aedes albopictus*, and *Culex quinquefasciatus* mosquitoes ompetence to Oropouche virus infection. Viruses 13(5): 755. 10.3390/v13050755 PMC8145018 33923055

[18] McGregor BL Connelly CR Kenney JL, 2021. Infection, dissemination, and transmission potential of North American *Culex quinquefasciatus, Culex tarsali*s, and *Culicoides sonorensis* for Oropouche virus. Viruses 13(2): 226. 10.3390/v13020226 PMC7912852 33540546

[19] Payne AF Stout J Dumoulin P Locksmith T Heberlein LA Mitchell M Rodriguez-Hilario A Dupuis AP 2nd Ciota AT, 2025. Lack of competence of US mosquito species for circulating Oropouche virus. Emerg Infect Dis 31(3): 619–621. 10.3201/eid3103.241886 PMC11878313 39837096

[20] de Mendonça SF, , 2025. *Oropouche orthobunyavirus* in urban mosquitoes: Vector competence, coinfection, and immune system activation in *Aedes aegypti*. Viruses 17(4): 492. 10.3390/v17040492 PMC12031340 40284935

[21] Jansen S Lühken R Höller P Mack A Lange U Jöst H Becker N Heitmann A, 2025. Risk assessment of Oropouche virus transmission by mosquitoes in Europe. J Infect Dis 232(6): e916–e919. 10.1093/infdis/jiaf356 PMC12718038 40626677

[22] Kim D Stenn TMS Dittman SM Sanchez YL Addae CA Torres LJR Buckner EA Burkett-Cadena ND Alto BW, 2025. Anthropophagic Florida mosquito species are poor vectors of prototype and emerging strains of Oropouche virus. PLoS Negl Trop Dis 19(12): e0013755. 10.1371/journal.pntd.0013755 PMC12680353 41325465

[23] Mancuso E, , 2025. Assessing the vector competence of Italian *Culex pipiens* and *Aedes albopictus* mosquitoes for the re-emerging Oropouche virus. Parasit Vectors 18(1): 268. 10.1186/s13071-025-06912-x PMC12235843 40629438

[24] Lainhart W Bickersmith SA Moreno M Rios CT Vinetz JM Conn JE, 2015. Changes in genetic diversity from field to laboratory during colonization of *Anopheles darlingi* Root (Diptera: Culicidae). Am J Trop Med Hyg 93(5): 998–1001. 10.4269/ajtmh.15-0336 PMC4703261 26283742

[25] LaReau JC Hyde J Brackney DE Steven B, 2023. Introducing an environmental microbiome to axenic *Aedes aegypti* mosquitoes documents bacterial responses to a blood meal. Appl Environ Microbiol 89(12): e0095923. 10.1128/aem.00959-23 PMC10734439 38014951

[26] Kilpatrick AM Fonseca DM Ebel GD Reddy MR Kramer LD, 2010. Spatial and temporal variation in vector competence of *Culex pipiens* and *Cx. restuans* mosquitoes for West Nile virus. Am J Trop Med Hyg 83(3): 607–613. 10.4269/ajtmh.2010.10-0005 PMC2929059 20810828

[27] Vaidyanathan R Scott TW, 2007. Geographic variation in vector competence for West Nile virus in the *Culex pipiens* (Diptera: Culicidae) complex in California. Vector Borne Zoonotic Dis 7(2): 193–198. 10.1089/vbz.2006.0589 17627438

[28] Darsie RF Jr Ward RA, 2005. Identification and Geographical Distribution of the Mosquitoes of North America, North of Mexico. 3rd ed. Gainesville, FL: University Press of Florida.

[29] Bhujel P Saha D, 2023. Rearing protocol for *Culex quinquefasciatus*. Sci Vis 2023: 61–65.

[30] Beaty BJ Calisher CH Shope RE, 1989. Arboviruses. Schmidt NJ Emmons RW, eds. Diagnostic Procedures for Viral Rickettsial and Chlamydial Infections. 6th ed. Washington, D.C.: American Public Health Association.

[31] Naveca FG Nascimento VAD Souza VC Nunes BTD Rodrigues DSG Vasconcelos P, 2017. Multiplexed reverse transcription real-time polymerase chain reaction for simultaneous detection of Mayaro, Oropouche, and Oropouche-like viruses. Mem Inst Oswaldo Cruz 112(7): 510–513. 10.1590/0074-02760160062 PMC5452489 28591313

[32] Ciota AT Bialosuknia SM Zink SD Brecher M Ehrbar DJ Morrissette MN Kramer LD, 2017. Effects of Zika virus strain and *Aedes* mosquito species on vector competence. Emerg Infect Dis 23(7): 1110–1117. 10.3201/eid2307.161633 PMC5512477 28430564

[33] Andreadis TG, 2012. The contribution of *Culex pipiens* complex mosquitoes to transmission and persistence of West Nile virus in North America. J Am Mosq Control Assoc 28(4 Suppl): 137–151. 10.2987/8756-971X-28.4s.137 23401954

